# Predictors of Weight Loss Maintenance following an Insurance-Sponsored Weight Management Program

**DOI:** 10.1155/2014/736080

**Published:** 2014-03-11

**Authors:** Christiaan G. Abildso, Olivier Schmid, Megan Byrd, Sam Zizzi, Alessandro Quartiroli, Sean J. Fitzpatrick

**Affiliations:** ^1^Department of Social and Behavioral Sciences, West Virginia University School of Public Health, P.O. Box 9190, Morgantown, WV, 26506-9190, USA; ^2^Institute of Sport Science, University of Bern, 3012 Bern, Switzerland; ^3^College of Physical Activity and Sport Sciences, West Virginia University, Morgantown, WV 26506-6116, USA; ^4^Department of Psychology, University of Wisconsin-La Crosse, La Crosse, WI 54601, USA; ^5^College of Graduate and Professional Studies, John F. Kennedy University, Pleasant Hill, CA 94523-4817, USA

## Abstract

Intentional weight loss among overweight and obese adults (body mass index ≥ 25 kg/m^2^) is associated with numerous health benefits, but weight loss maintenance (WLM) following participation in weight management programming has proven to be elusive. Many individuals attempting to lose weight join formal programs, especially women, but these programs vary widely in focus, as do postprogram weight regain results. We surveyed 2,106 former participants in a community-based, insurance-sponsored weight management program in the United States to identify the pre, during, and post-intervention behavioral and psychosocial factors that lead to successful WLM. Of 835 survey respondents (39.6% response rate), 450 met criteria for inclusion in this study. Logistic regression analyses suggest that interventionists should assess and discuss weight loss and behavior change perceptions early in a program. However, in developing maintenance plans later in a program, attention should shift to behaviors, such as weekly weighing, limiting snacking in the evening, limiting portion sizes, and being physically active every day.

## 1. Introduction

Intentional weight loss among overweight and obese adults (body mass index ≥ 25 kg/m^2^) is associated with numerous health benefits. Reviews of the literature suggest that diet-plus-physical activity weight loss interventions produce greater weight losses than diet-only interventions [1, 2]. However, weight loss maintenance (WLM) continues to be the Achilles heel of many such interventions, with postprogram weight regains in diet-plus-physical activity lifestyle interventions of generally 50% by one year after intervention [1, 3]. A systematic review of studies published between 1966 and 2008 suggests that 2 to 54% of lifestyle intervention participants achieve intentional WLM [3, 4]. This wide variation in rate of “successful losers” is primarily accounted for by inconsistent definitions of WLM, which is commonly conceptualized as a combination of achieving a specified minimum weight loss and sustaining it over a certain period of time [3, 4].

Various criteria have been used in the literature to determine successful WLM, including the duration of the active weight loss and weight maintenance phases, the amount of weight loss during the active and maintenance phases, the types of interventions, and the times of assessment. Maintaining a 5–10% weight loss has been shown to have clinically significant health benefits [5, 6] and an increase by 50%, the likelihood of successful maintenance over five years [7]. An even lower amount of initial weight loss may have additional benefits for WLM, as higher amounts of weight loss do not improve the prediction of WLM [3] and may be associated with weight regain, cycling, yo-yo dieting, and ill health [8–10]. Stevens and colleagues [11] further recommended that a weight change of ±3% is to be considered weight maintenance, weight changes ranging from 3% to 5% is to be considered small weight fluctuations, and weight loss of >5% is to be considered clinically significant. Despite the lack of definitional consensus, adopting more inclusive definitions of weight loss maintenance that allows for some regain following loss appears to provide participants with the most health benefits [4, 7].

Methodological concerns notwithstanding, a variety of behavioral and psychosocial predictors have been identified to account for successful WLM. Individuals who have successfully achieved self-directed WLM have been found to be more physically active during their period of weight loss than their unsuccessful peers [12, 13]. In addition, eating behaviors such as consuming breakfast regularly, reducing portion size, and limiting snacking have been found to predict lower caloric intake [14–17]. Self-monitoring strategies, such as keeping a food and exercise log and frequent weighing, have also been found to be critical for WLM [7, 13, 14, 18, 19]. Psychosocial predictors have commonly included receiving social support from a weight maintenance group or friends, but the beneficial impact of spousal participation has remained inconsistent [4]. In addition to the findings about social support, autonomy and self-reliance have also predicted successful WLM [20].

National Weight Control Registry research suggests that the majority of successful weight loss maintainers, especially women, participate in a formal program to achieve initial weight loss [13] and keep using the behavior change strategies learned during the interventions after the intervention is completed [7]. Large corporations and health insurance companies worldwide have a key role to play in incentivizing participation in weight management programming and have started investing in such initiatives [21, 22]. Therefore, defining the most effective in-program strategies to prevent weight regain following weight loss is critical for weight management interventions [3]. The purpose of this study was to identify the pre-, during, and postintervention behavioral and psychosocial factors that lead to successful WLM following participation in an insurance-sponsored diet-plus-physical activity community-based intervention.

## 2. Methods

### 2.1. Participants

West Virginia Public Employees Insurance Agency (PEIA) members that enrolled in PEIA's weight management program (WMP) benefit between April 1, 2005, and June 30, 2008, were recruited to complete a program evaluation and postprogram health behavior survey in February 2009 (*N* = 2,106). The enrollment dates were chosen to ensure that all participants contacted had the time to have completed at least six months of the WMP by the time study recruitment began. A full evaluation and details of the WMP [23, 24] are available. Briefly, the WMP is an insurance benefit that provides access to exercise and nutrition professionals for a small monthly copayment at private exercise facilities with intervention services decreasing as participants progress through the program of up to two years (see Table 1 for details). Facilities are reimbursed by PEIA for services provided using a predetermined fee schedule, and participant progress is tracked by care management nurses. A 12-pound weight loss is expected of participants by the end of month 3 of the WMP. Otherwise, no weight loss, calorie intake, or physical activity goal is mandated or strictly enforced. Participants may also be removed from the program for noncompliance with the following behavioral expectations: exercising at their site at least twice per week; turning in food logs periodically; attending appointments with the exercise physiologist, registered dietitian, and personal trainer; and having monthly body measurements taken by site staff. Professional exercise and nutrition services are provided following relevant guidelines for weight loss and maintenance (e.g., American College of Sports Medicine, American Dietetic Association).

**Figure 1:**
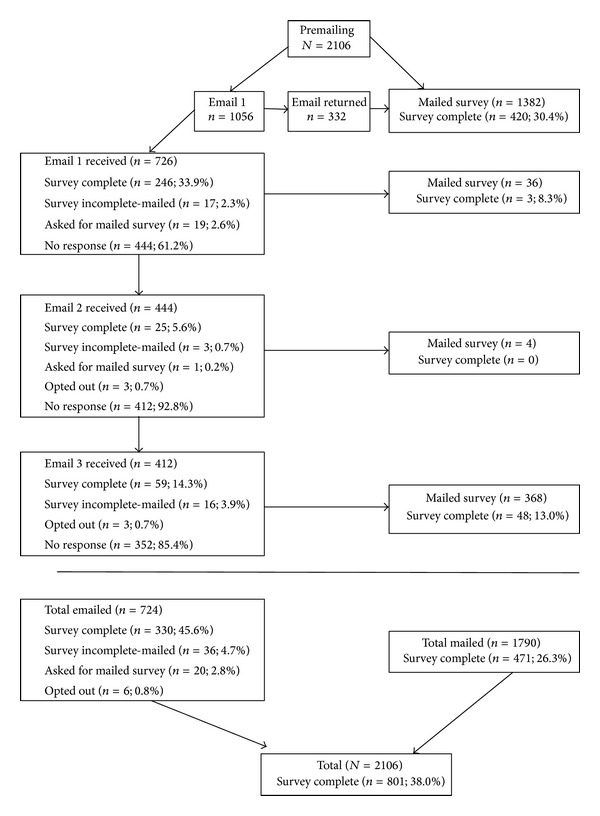
Sample phases and response rates.

This study was approved by the West Virginia University Institutional Review Board. Using a modified version of Dillman's [25] recruiting method; eligible participants (*N* = 2,106) were contacted by mail and/or email up to five times over the course of six weeks to complete a program evaluation and postprogram health behavior survey (see Figure 1). All 2,106 eligible participants were sent a letter in February 2009 notifying them that a survey would be forthcoming and were sent a follow-up by email (*n* = 1,056) or mail (*n* = 1,050) with a link to, or a hard copy of, the survey depending on the availability of a valid email address. Those with a valid email address were sent two reminders before being mailed a hard copy of the survey. Surveys were mailed to those with invalid email addresses (*n* = 332). These participants, and those without an email address, were sent a follow-up letter within three weeks of receiving the hard copy of the survey if they had not filled out and returned the survey. To encourage participation, the opportunity to enter a random drawing for 100 recipients to receive a free health-related book was offered.

### 2.2. Instrumentation

Participants were asked to complete a program evaluation survey containing a mix of open-ended and closed-ended items in sections categorized chronologically as they related to the WMP (i.e., pre-, during, and postprogram). Each section had a prompt to ensure the respondent was evaluating the correct time period (e.g., “The next set of questions asks you about your participation in the Program”). The survey sections and items pertinent to this study of WLM are described in detail below.

#### 2.2.1. Preprogram Factors

The first section of the survey contained items assessing preprogram factors including demographic information, physical activity and weight loss history, and bariatric surgery intention.

*Demographic Information*. Survey items assessed demographic information including age, gender, marital status, and number of dependents in the home (i.e., caregiver status). Based on response distribution, age was categorized as* <45 years*,* 45–54.9 years*, or* ≥55 years*; marital status was categorized as* married* or* unmarried* (single/divorced/widowed). Caregiver status was determined by using the number of dependents in the home item to categorize the respondent as a* caregiver* (one or more dependents in the home) or* noncaregiver* (zero dependents in the home). Race and employment statuses were not used as predictors because over 90% of participants are white (reflective of the population of West Virginia) and full-time employees eligible for this insurance benefit. 

*Physical Activity and Weight Loss History*. Physical activity was determined using condensed versions of Behavioral Risk Factor Surveillance System (BRFSS) physical activity module items [26]. Separate items queried participants to retrospectively assess the number of days in a usual week that they did 30 or more minutes of moderate physical activity (MPA) and 20 or more minutes of vigorous physical activity (VPA) in the six months prior to entering the WMP. Because of the retrospective nature of this item, responses were categorized into* sedentary* (zero MPA and VPA) versus* any activity* (nonzero MPA or VPA). The number of weight loss attempts was used to assess weight loss history. Responses were categorized into quartiles for analysis (*<5, 5–9, 10–19,* or* ≥20*). Participants were also asked if they were considering bariatric surgery before joining the WMP (*yes/no*).

#### 2.2.2. In-Program Factors

In the second section of the survey, respondents were asked to evaluate in-program factors including perceptions of weight loss, effort, and success and difficulty of health behavior change and maintenance as they progressed through Phase I of the program (months 1–3) and beyond. 

*Perception of Weight Loss, Effort, and Success*. To understand perception of initial weight loss, participants were asked to rate their weight loss during Phase I as* Excellent*,* Good*,* Acceptable*,* Poor*, or* Disappointing*. This was condensed based on response distribution as* Excellent/Good*,* Acceptable*, or* Poor/Disappointing*. In addition, they were asked to provide numerical ratings for their perceived effort during Phase I from 0 (*least*) to 100 (*most*) and success during Phase 1 from 0 (*worst*) to 100 (*most*). Based on prior research [27], these responses were compared and condensed into three categories for analysis (*success > effort*,* success = effort*, or* success < effort*). 

*Perceived Difficulty of Health Behavior Change and Maintenance*. Perceived difficulty of initial health behavior change was assessed using multiple items to rate the difficulty of losing weight, changing diet routine, and starting an exercise routine during Phase I on a six-point scale from 1 (*extremely easy*) to 6 (*extremely difficult*). Perceived difficulty of maintaining these health behavior changes was assessed similarly, using items to rate the difficulty of sticking with diet changes and continuing an exercise routine beyond Phase I on a six-point scale from 1 (*extremely easy*) to 6 (*extremely difficult*). Responses were split at the midpoint to dichotomize the variables to* easy *(responses 1–3) versus* difficult* (responses 4–6).

#### 2.2.3. Postprogram Factors

In the final section of the survey, we assessed postprogram factors (i.e., current health behaviors). These included current physical activity level, weight management behaviors, food management strategies, and current height and weight. 

*Current Physical Activity*. Physical activity was assessed using condensed versions of items from the physical activity module of the BRFSS [26]. Separate items asked the respondent to assess the number of days in a usual week; they did 30 or more minutes of MPA and 20 or more minutes of VPA. Responses were categorized into* sedentary* (zero MPA and VPA),* insufficiently active *(not meeting MPA or VPA guidelines), or* sufficiently active* (meeting MPA and/or VPA guidelines). 

*Weight Management Behaviors*. Behaviors associated with WLM were assessed in the instrument, including frequency of self-weighing (*never*,* <1 time per week*,* weekly but not daily*,* and daily*), current method of weight loss (*not currently trying to lose weight*,* activity or diet alone*,* and activity and diet in combination*), frequency of eating breakfast (*daily*,* not daily*), logging physical activity (*yes*,* no*), and currently exercising at a gym, or WMP facility (*yes*,* no*). 

*Food Management Behaviors*. Seven behavioral food strategies to maintain weight associated with WLM were also assessed in the instrument by asking the respondent to endorse which strategies they were currently using. These strategies included counting calories, limiting the amount of fat consumed, eating out less often, limiting portion size at meals, keeping a food log or journal, limiting soda and sweetened drinks, and limiting snacking in the evening. All were coded as* yes*/*no* based on respondent endorsement or not.

### 2.3. Weight Data, Length of Time in Program, and Length of Time after Program

Fitness and exercise professionals at facilities measure participant data monthly, including height, weight, and body mass index (BMI). Each site determines its measurement protocols on the basis of available instruments and staff training. While protocols and instrumentation may vary across facilities, they do not vary within facilities over time. Data are entered into a secure database from which data were extracted for the current study. Baseline and final program measurements were used to calculate baseline BMI (*25–29.9, 30–34.9, 35–39.9, and ≥40 *kg/m^2^) and percentage of baseline weight lost during the program. In-program weight loss was categorized as* clinically significant (≥5%) *or* nonclinically significant (<5%)* [28–30]. Further, because each data point has a date associated with it, these data were used to calculate the length of time each participant remained in the program. Six months is generally the point at which habits are formed [31], the common length of weight management interventions, and the point at which weight loss peaks in these interventions [32–34]. Further, the WMP moves to a minimal “maintenance” intervention period (Phase III) after the 12th month. Thus, length of time in the program was classified as* ≤6 months*,* >6–12 months*, or* >12 months*. Lastly, the final measurement date and the date of the survey response were used to calculate the postprogram time, classified as* ≤6 months*,* >6–12 months*,* >12–24 months*, or* >24 months*.

### 2.4. Analyses

Statistical analyses were conducted using SPSS version 19.0. Comparisons of successful maintainers (SM) and unsuccessful maintainers (UM) of weight loss were conducted using independent samples *t*-tests for continuous dependent variables or chi-square analyses for categorical dependent variables. Forward stepwise logistic regression analysis, an effective exploratory technique [35, 36], was conducted to identify the predictors of WLM, our outcome of interest. We operationalized WLM as any participant that met the following criteria: (a) lost any amount of weight during the WMP, (b) maintained that weight loss or regained <4% of postprogram weight during the time from program end to survey completion, and (c) achieved overall weight loss during the preprogram to survey completion time point. As called for in recent literature, this is a very inclusive operationalization of WLM which allows for moderate short-term losses that may lead to greater losses over the longer term, excludes extreme weight loss changes, and allows for minimal regain postintervention [4, 11].

Four regression models were run to determine factors to include in a final predictive model. Repeated contrasts were used for each predictor variable in each model. This method compares each category of a predictor (except the first) to the previous category. Thus, contrasts include categories 1 versus 2, 2 versus 3, and 3 versus 4, rather than the simple contrasts of categories 1 versus 2, 1 versus 3, 1 versus 4, and so on. This allows for pinpointing specific frequencies of behaviors, such as self-weighing and amount of PA, predictive of WLM.

Model A (preprogram) included four demographic factors (age, gender, marital status, and caregiver status), physical activity level, whether weight loss surgery was being considered or not, and objectively measured baseline BMI. Model B (in-program) consisted of nine factors, including perception of weight loss, difference between perceived effort and success, five perceived difficulties of health behavior change items, and objectively measured percentage weight loss and length of time in the program. Model C (postprogram 1) included seven factors, specifically length of time from program end to survey completion date, frequency of self-weighing, current method of weight loss, frequency of eating breakfast, logging physical activity, and currently exercising at a gym or WMP facility. Model D (postprogram 2) consisted of seven food management behavioral factors including counting calories, limiting the amount of fat consumed, eating out less often, limiting portion size at meals, keeping a food log or journal, limiting soda and sweetened drinks, and limiting snacking in the evening.

Predictors significant at the *P* < 0.05 level from Models A–D were included in the final model. Odds ratios (ORs) and 95% confidence intervals (CI) are reported for successfully achieving WLM as operationalized in this study. Because we used repeated contrasts for each predictor variable, ORs should be interpreted as the change in the likelihood of being a successful maintainer (SM) that results in a one-unit increase in the predictor variable. Thus an OR > 1 should be interpreted as an increase, and OR < 1 should be interpreted as a decrease, in the likelihood of being a SM with a one-unit increase in the predictor.

## 3. Results

### 3.1. Response and Baseline Data

A total of 835 surveys were received (39.6% response rate), 801 of which were complete. From these, 351 completed surveys were removed because they did not have a weight measurement following baseline (*n* = 26), had a baseline BMI <25 kg/m^2^ (*n* = 3), became pregnant during the program (*n* = 4), had bariatric surgery postprogram (*n* = 7), did not report a current weight (*n* = 21), were still active in the program when they completed the survey (*n* = 154), were <1 month after program at the time of survey completion (*n* = 39), were duplicate entries from the same individual across survey platforms (*n* = 2), or had gained weight during the program (*n* = 95). The resulting analytic sample size was *N* = 450. Our sample was largely females (81.1%), married (74.9%), and 45 years or older (74.2%).

Nearly half of the respondents were successful at WLM (*n* = 210, 46.7%). Independent samples *t*-tests showed that SM and UM did not achieve significantly different percentage weight loss during the program (6.4% versus 7.2%; *P* = 0.157) but did achieve significantly different weight change after program and overall from preprogram to current time (*P* < 0.001). In fact, SM lost 2.4% of end program weight and* lost* 8.6% of preprogram weight overall, a clinically significant loss [37]. In comparison, UM* gained *9.6% postprogram and* gained *1.5% from preprogram. A greater percentage of SM were meeting PA guidelines after program than UM (43.8% versus 24.7%), and fewer SM than UM were insufficiently active (41.3% versus 51.5%) or sedentary (14.9% versus 23.8%) after program (*χ* ^2^ = 19.000; *P* < 0.001).

### 3.2. Predictors of WLM

Items included in, and results of, Models A–D are presented in Tables 2, 3, 4, and 5. Please note that the size of the analytic sample in each model varies because SPSS performs a listwise deletion of missing data when running logistic regression. Thus, if there is a missing value for any variable in the model, the entire case is excluded from the analysis. Tables 2–5 present all potential predictor variables in the order in which the repeated contrasts were conducted. The Wald chi-square statistic, which indicates whether **β** for each variable is significantly different than zero and the variable is a significant predictor of weight loss maintenance [35], is reported for all variables, but an OR is only reported for significant predictors. Larger values of the Wald statistic indicate a variable more likely to be a significant predictor of the outcome.

Preprogram physical activity level was the only significant predictor of WLM from regression Model A, with SM more likely to have been getting any physical activity before WMP than UM (OR = 1.62, 95% CI = 1.05–2.51). Regression Model B revealed that respondents completing >6–12 months of the program were* less likely* to be a SM than those that completed at least 12 months (OR = 0.55, 95% CI = 0.32–0.94); respondents rating Phase I weight loss as acceptable were more likely to be SM than respondents rating weight loss as good or excellent (OR = 2.19, 95% CI = 1.35–3.58); respondents indicating it was easy to stick with diet changes (OR = 2.24, 95% CI = 1.25–4.00) and easy to continue a regular exercise routine (OR = 2.15, 95% CI = 1.25–3.71) were more likely to be SM than those rating those changes as difficult. Significant predictors of SM from Model C indicate that respondents >6–12 months after program were more likely be SM than respondents >12–24 months after program (OR = 2.97, 95% CI = 1.66–5.34); respondents insufficiently active were* less likely* than their sufficiently active peers to be SM (OR = 0.46, 95% CI = 0.28–0.76); and respondents weighing themselves less than once per week were* less likely* to achieve SM than respondents weighing themselves at least once per week but not daily (OR = 0.33, 95% CI = 0.19–0.56). Model D produced two food management behaviors predictive of SM: limiting portion sizes (OR = 2.32, 95% CI = 1.55–3.46) and limiting snacking in the evening (OR = 1.71, 95% CI = 1.15–2.54).

In the final model, six factors significantly predicted SM (see Table 6 for details), including being >12–24 months after program compared to >24 months after program (OR = 1.81, 95% CI = 1.02–3.23); being >6–12 months after program compared to >12–24 months after program (OR = 2.67, 95% CI = 1.48–4.84); self-weighing less than once per week compared with weekly but not daily (OR = 0.39, 95% CI = 0.23–0.66); limiting snacking in the evening (OR = 2.12, 95% CI = 1.33–3.38); limiting portion sizes (OR = 1.99, 95% CI = 1.25–3.19); rating Phase I weight loss as acceptable compared with good/excellent (OR = 2.21, 95% CI = 1.28–3.80); and perceiving it to be easy to continue a regular exercise routine as compared with difficult (OR = 2.40, 95% CI = 1.42–4.06).

## 4. Discussion

In agreement with published research [38], results from this study suggest that the likelihood of successfully maintaining weight loss diminishes over time, peaking in our survey respondents in the 6–12-month postprogram timeframe and decreasing in a stepwise fashion over time.

Preprogram physical activity level significantly predicted WLM, but only in the regression model that included preprogram predictors. However, results from the comprehensive predictor model of our study suggest no significant preprogram predictors of WLM. This is a positive finding from a population-based perspective in that it shows the program works similarly in a real-world environment with people of varying demographic characteristics, weight loss histories, BMI, and PA.

Respondents who perceived their early program weight loss as* acceptable* were more than twice as likely to achieve WLM as those who rated their weight loss as* good or excellent*. This finding is similar to prior research that suggests that unrealistic weight loss expectations are associated with dropout from weight management programs [39–41] and that program completers achieve results that closely match preprogram expectations [42]. Interventionists should frequently assess and discuss perception of in-program weight loss, especially early, to make sure that participants perceive that they are gradually meeting modest, realistic weight loss goals. In contrast, individuals that perceive weight loss as* good* or* excellent* may believe the behavior change process to be easy, underestimating the vigilance and cognitive restraint [43] needed to maintain such changes, leading to overconfidence, dropout, and weight regain/cycling.

The perception that maintaining a regular exercise routine was* easy* (compared with* difficult*) was predictive of WLM, suggesting another target for intervention. This confirms other research findings [4] that an individual's self-efficacy, or belief in their ability to accomplish a behavior [44] - in this case exercise—is important for sustaining weight loss. SM were more likely to meet PA guidelines. However, contrary to prior research [4, 7, 45], postprogram PA level was not predictive of WLM in the final regression model though our measurement of PA was dissimilar to prior studies.

Postprogram behaviors were predictive of WLM and should be considered as education components that are incorporated later in WMPs, reinforced with participants upon completing WMPs, and targeted for “booster” postprogram interventions. These included weekly weighing, limiting snacking in the evening, and limiting portion sizes. In concordance with prior studies [7], self-weighing was predictive of SM, specifically self-weighing at least once per week as compared to less frequent weighing. In addition, it would be beneficial to work with participants to develop long-term strategies for limiting evening snacking (e.g., brushing teeth immediately after dinner, drinking water instead of snacking, and limiting the availability of snacks in the home) and limiting portion sizes (e.g., using portion control dishware, learning to measure portions accurately, and immediately putting half of a dinner in a to-go box when eating out). These strategies can easily be gleaned from surveys or interviews with individuals successful at WLM.

It is critical that the findings of the current study be viewed in the appropriate context. The program we evaluated is a community-based, public insurance benefit for working adults in a rural state in the USA (West Virginia) that has some of the highest rates of chronic disease in the country. Its development was informed by evidence-based programs (i.e., Diabetes Prevention Program) but adapted to be contextually appropriate and sustainable. Recent reviews and meta-analyses of randomized, controlled trials (e.g., [46]) have elucidated findings from such work. Thus, context-specific findings of our evaluation that are incongruent with others' (e.g., nonsignificant PA-WLM relationship) may be a result of the different setting, intervention approach, and/or assessment methods of our work from that of RCTs, and the limitations of our study are discussed in what follows. First, we used an inclusive definition of WLM as the outcome variable in agreement with recent recommendations [4, 11]. A more conservative definition of WLM in weight loss amount and/or length of maintenance such as those used by the NWCR and IOM [7, 47] may have yielded different results. Second, preprogram factors were assessed retrospectively. Though half of the respondents began the program within two years of this project, they may have begun up to four years prior to the survey. This time delay may have limited the ability of respondents to accurately assess some variables. To address this limitation, we categorized preprogram PA responses as* none* or* some* because it is likely that people were able to recall the difference between doing any or no PA rather than specific minutes of PA in a week. Third, the majority of predictor variables and current weight used to categorize WLM were assessed via a self-report. Such subjective reports of perceptions are relevant as intervention targets, but self-report of weight and PA has inherent weaknesses. Further, some items in our evaluation survey were taken from the current evidence base and are specific to this evaluation but not yet validated. Additionally, generalizability of the findings is limited to participants in similar insurance-sponsored programs because a large portion of survey respondents are white, full-time employed, and married women over 45 years old. However, these demographics are similar to studies related to other formal programs and the NWCR sample [13]. Also, our response rate (39.6%) may be considered less than ideal, but it is consistent with a meta-analysis of survey research (39.6%) [48]. Sampling error was controlled by inviting all members of the population to complete the survey, and nonresponse error appears low because responders did not differ from nonresponders in any of the key variables such as BMI, program completion, or 5% weight loss rate.

## 5. Conclusions

Despite the aforementioned limitations, modifiable perceptions and behaviors predictive of WLM that could be targets of future interventions were identified in this study. Many SM in the current study were able to achieve and maintain a clinically meaningful amount of weight loss, providing valuable guidance for other programs. The results suggest that weight loss interventionists should change intervention targets as participants move through a WMP, in concordance with the shift from cognitive to behavioral processes of change as individuals progress through the stages of change [31]. Early in a program, interventionists may want to assess and discuss weight loss and behavior change perceptions as these may reflect participant self-efficacy and, ultimately, participant retention. As individuals progress through a program and shift toward maintenance of weight loss, interventionists are encouraged to focus attention on behaviors in developing maintenance plans, such as weekly weighing, limiting snacking in the evening, limiting portion sizes, and being physically active every day.

## Figures and Tables

**Table 1:** Minutes of services per participant and monthly reimbursement made by the insurer during the weight management program.

Service	Phase I (months 1–3)			Phase II (months 4–12)									Phase III (months 13–24)	
	M1	M2	M3	M4	M5	M6	M7	M8	M9	M10	M11	M12	M13	M14–24
Registered dietitian	60	—	60	—	—	30	—	—	30	—	—	—	60	—
Fitness assessment	60	—	60	—	—	30	—	—	30	—	—	—	60	—
Personal training	30	30	30	15	15	15	15	15	15	15	15	15	15	15/mo
Member copayment	$45	$45	$45	$14	$14	$14	$14	$14	$14	$14	$14	$14	$25 max^a^	$25 max^a^
Agency payment to facility	$246.67	$246.67	$246.67	$32	$32	$32	$32	$32	$32	$32	$32	$32	$25 max^a^	$25 max^a^

a: member copayment (and insurance agency payment to the facility) during months 13–24 is one-half of the facility's maximum published private membership fee up to a maximum of $50.

**Table 2:** Preprogram predictors of weight loss maintenance (*N* = 404)—Model A.

	*n*	*β*	Wald *χ* ^2^	OR (95% CI)
Marital status				
Single/divorced/widowed	102		—	
Married	302		0.001	
Gender				
Female	327		—	
Male	77		0.19	
Caregiver				
No	248		—	
Yes	156		0.25	
Considering bariatric surgery				
No	312		—	
Yes	92		0.24	
Preprogram MVPA				
None (sedentary)	120		—	1.00
Any activity	284	0.48	4.69	1.62 (1.05–2.51)*
Age at the program start, years				
55+	134		—	
45–54.9	164		2.84	
<45	106		0.003	
Baseline body mass index, kg/m^2^				
Obese III (40+)	132		—	
Obese II (35–39.9)	96		0.88	
Obese I (30–34.9)	140		0.72	
Overweight (25–29.9)	36		1.01	
Weight loss attempts				
≥20	137		—	
10–19	107		2.32	
5–9	73		0.02	
<5	87		0.31	

Note: MVPA: moderate-to-vigorous physical activity *P < 0.05; **P < 0.01; ***P < 0.001.

The Wald *χ* ^2^ statistic, which indicates whether *β* for each variable is significantly different than zero, and the variable is a significant predictor of weight loss maintenance and is reported for all variables, but an OR is only reported for significant predictors. Each variable is presented in the order in which the repeated contrasts were conducted. Thus within each variable, each level moving down the rows of the table should be compared with the level of the variable in the row immediately above it. Thus, ORs should be interpreted as the change in the likelihood of being a successful maintainer (SM) that results in a one-unit increase in the predictor variable represented by a move one row down in the table.

**Table 3:** In-program predictors of weight loss maintenance (*N* = 428)—Model B.

	*n*	*β*	Wald *χ* ^2^	OR (95% CI)
In-program weight loss				
Not clinically significant (<5%)	208		—	
Clinically significant (≥5%)	220		3.45	
Months in the program				
>12	94		—	1.00
>6–12	182	−0.60	4.73	0.55 (0.32–0.94)*
≤6	152	−0.09	0.14	0.92 (0.58–1.46)
Perceived Phase I weight loss				
Good/excellent	228		—	1.00
Acceptable	110	0.79	9.91	2.19 (1.35–3.58)**
Poor/disappointing	90	−0.41	1.85	0.67 (0.37–1.20)
Perceived Phase I effort/success balance				
Success < effort	179		—	
Success = effort	188		0.27	
Success > effort	61		0.88	
Perceived difficulty to				
Start an exercise routine				
Difficult to extremely difficult	244		—	
Easy to extremely easy	184		1.39	
Change diet				
Difficult to extremely difficult	258		—	
Easy to extremely easy	170		1.91	
Lose weight				
Difficult to extremely difficult	267		—	
Easy to extremely easy	161		2.99	
Continue regular exercise routine				
Difficult to extremely difficult	313		—	1.00
Easy to extremely easy	115	0.77	7.56	2.15 (1.25–3.71)**
Stick with diet changes				
Difficult to extremely difficult	331		—	1.00
Easy to extremely easy	97	0.80	7.37	2.24 (1.25–4.00)**

Note: *P < 0.05; **P < 0.01; ***P < 0.001.

The Wald *χ* ^2^ statistic, which indicates whether *β* for each variable is significantly different than zero, and the variable is a significant predictor of weight loss maintenance and is reported for all variables, but an OR is only reported for significant predictors. Each variable is presented in the order in which the repeated contrasts were conducted. Thus, within each variable, each level moving down the rows of the table should be compared with the level of the variable in the row immediately above it. Thus, ORs should be interpreted as the change in the likelihood of being a successful maintainer (SM) that results in a one-unit increase in the predictor variable represented by a move one row down in the table.

**Table 4:** Postprogram predictors of weight loss maintenance (*N* = 404)—Model C.

	*n*	*β*	Wald *χ* ^2^	OR (95% CI)
Months postprogram				
>24	112		—	
>12–24	135	0.24	0.69	1.27 (0.72–2.21)
>6–12	92	1.09	13.33	2.97 (1.66–5.34)***
≤6	65	0.48	1.68	1.61 (0.78–3.31)
Self-weighing frequency				
At least once every day	39		—	
At least once per week but not daily	190	−0.28	0.52	0.76 (0.35–1.62)
Less than once per week	120	−1.11	16.51	0.33 (0.19–0.56)***
Never	55	0.42	1.27	1.53 (0.73–3.19)
Current weight loss method				
Using both physical activity and diet	209		—	
Using physical activity or diet alone	126		1.41	
Not currently trying to lose weight	69		0.004	
Current level of physical activity				
Meeting guidelines	133		—	
Insufficiently active	191	−0.77	9.37	0.46 (0.28–0.76)**
Sedentary	80	0.06	0.05	1.07 (0.59–1.93)
Eating breakfast daily				
No	226		—	
Yes	178		0.03	
Keeping a physical activity log				
No	363		—	
Yes	41		0.94	
Currently exercising at a gym or WMP site				
No	293		—	
Yes	111		1.22	

Note: WMP: weight management program. *P < 0.05; **P < 0.01; ***P < 0.001.

The Wald *χ* ^2^ statistic, which indicates whether *β* for each variable is significantly different than zero, and the variable is a significant predictor of weight loss maintenance and is reported for all variables, but an OR is only reported for significant predictors. Each variable is presented in the order in which the repeated contrasts were conducted. Thus within each variable, each level moving down the rows of the table should be compared with the level of the variable in the row immediately above it. Thus, ORs should be interpreted as the change in the likelihood of being a successful maintainer (SM) that results in a one-unit increase in the predictor variable represented by a move one row down in the table.

**Table 5:** Postprogram predictors of weight loss maintenance (*N* = 450)—Model D.

	*n*	*β*	Wald *χ* ^2^	OR (95% CI)
Limiting snacking in the evening				
No	197		—	
Yes	253	0.53	6.90	1.71 (1.15–2.54)**
Limiting amount of fat consumed				
No	233		—	
Yes	217		0.42	
Eating out less often				
No	242		—	
Yes	208		2.11	
Limiting portion size at meals				
No	188		—	
Yes	262	0.84	16.71	2.32 (1.55–3.46)***
Keeping a food log or journal				
No	348		—	
Yes	102		3.21	
Limiting soda or sweetened drinks				
No	173		—	
Yes	277		0.01	
Counting calories				
No	305		—	
Yes	145		0.89	

Note: *P < 0.05; **P < 0.01; ***P < 0.001.

The Wald *χ* ^2^ statistic, which indicates whether *β* for each variable is significantly different than zero, and the variable is a significant predictor of weight loss maintenance and is reported for all variables, but an OR is only reported for significant predictors. Each variable is presented in the order in which the repeated contrasts were conducted. Thus, within each variable, each level moving down the rows of the table should be compared with the level of the variable in the row immediately above it. Thus, ORs should be interpreted as the change in the likelihood of being a successful maintainer (SM) that results in a one-unit increase in the predictor variable represented by a move one row down in the table.

**Table 6:** Final model predicting weight loss maintenance (*N* = 428).

	*n*	*β*	Wald *χ* ^2^	OR (95% CI)
Preprogram MVPA				
None (sedentary)	130		—	
Any activity	298		0.05	
Perceived Phase I weight loss				
Good/excellent	227		—	1.00
Acceptable	112	0.79	8.17	2.21 (1.28–3.80)**
Poor/disappointing	89	−0.48	2.14	0.62 (0.33–1.18)
Months in the program				
>12	95		—	
>6–12	181		1.63	
≤6	152		0.05	
Perceived difficulty of sticking with diet changes				
Difficult to extremely difficult	331		—	
Easy to extremely easy	97		2.67	
Perceived difficulty of continuing exercise routine				
Difficult to extremely difficult	313		—	1.00
Easy to extremely easy	115	0.88	10.63	2.40 (1.42–4.06)***
Current level of physical activity				
Meeting guidelines	146		—	
Insufficiently active	200		0.76	
Sedentary	82		0.18	
Months after program				
>24	121		—	1.00
>12–24	145	0.59	4.04	1.81 (1.02–3.23)*
>6–12	98	0.98	10.60	2.67 (1.78–4.84)***
≤6	64	0.18	0.23	1.20 (0.57–2.54)
Self-weighing frequency				
At least once every day	36		—	1.00
At least once per week but not daily	207	−0.79	3.32	0.45 (0.19–1.06)
Less than once per week	127	−0.96	12.12	0.39 (0.23–0.66)***
Never	58	0.27	0.45	1.31 (0.59–2.91)
Limiting snacking in the evening				
No	188		—	1.00
Yes	240	0.75	9.87	2.12 (1.33–3.38)**
Limiting portion size at meals				
No	180		—	1.00
Yes	248	0.69	8.27	1.99 (1.25–3.19)**

Note: MVPA = moderate-to-vigorous physical activity. *P < 0.05; **P < 0.01; ***P < 0.001.

The Wald *χ* ^2^ statistic, which indicates whether *β* for each variable is significantly different than zero, and the variable is a significant predictor of weight loss maintenance and is reported for all variables, but an OR is only reported for significant predictors. Each variable is presented in the order in which the repeated contrasts were conducted. Thus within each variable, each level moving down the rows of the table should be compared with the level of the variable in the row immediately above it. Thus, ORs should be interpreted as the change in the likelihood of achieving weight loss maintenance that results in a one-unit increase in the predictor variable represented by a move one row down in the table.
